# Supplementary material for: Maternal and neonatal outcomes in patients with hepatitis C and intrahepatic cholestasis of pregnancy: The sum of the parts
Source: PLoS One. 2023 Oct 18;18(10):e0293030. doi: 10.1371/journal.pone.0293030 (PMC10584137; doi:10.1371/journal.pone.0293030)
Supplement: S2 Checklist — (DOCX) [file pone.0293030.s002.docx]

STROBE Statement—checklist of items that should be included in reports of observational studies

|  | Item No. | Recommendation | Page  No. | Relevant text from manuscript |
| --- | --- | --- | --- | --- |
| **Title and abstract** | 1 | (*a*) Indicate the study’s design with a commonly used term in the title or the abstract | 2 | “We conducted a retrospective cohort study of the Nationwide Readmissions Database…” |
|  |  | (*b*) Provide in the abstract an informative and balanced summary of what was done and what was found | 2-3 | See abstract |
| Introduction | | | |  |
| Background/rationale | 2 | Explain the scientific background and rationale for the investigation being reported | 4 | “There is an increased risk of ICP in HCV-infected pregnant patients, and those with ICP are at increased risk of more advanced HCV infection and higher HCV viral load [8-10]. While it is known that each condition is associated with an increased risk of adverse maternal and obstetric outcomes, there are currently no studies investigating the effects of HCV and ICP co-occurrence on outcomes.” |
| Objectives | 3 | State specific objectives, including any prespecified hypotheses | 4 | Our objective was to compare maternal outcomes between birthing individuals with HCV alone, ICP alone, and HCV and ICP together. Given that both diseases target the liver, it may be that these two diseases interact to worsen severe maternal morbidity, beyond the simple additive combination of the two (i.e., that they demonstrate synergy). |
| Methods | | | |  |
| Study design | 4 | Present key elements of study design early in the paper | 4 | “This retrospective cohort study was conducted using the Nationwide Readmissions Database (NRD), from the United States Agency for Healthcare Research and Quality’s Healthcare Cost and Utilization Project” |
| Setting | 5 | Describe the setting, locations, and relevant dates, including periods of recruitment, exposure, follow-up, and data collection | 5 | “The NRD is an all-payor administrative database designed to represent short-stay inpatient admissions in the United States. A total of 28 states participated in the NRD in 2020, including 59.7% of the population and 58.7% of hospitalizations”  “Deliveries at or beyond 24 weeks gestation with hospital discharges between 10/2015 and 12/2020 were included.” |
| Participants | 6 | (*a*) *Cohort study*—Give the eligibility criteria, and the sources and methods of selection of participants. Describe methods of follow-up  *Case-control study*—Give the eligibility criteria, and the sources and methods of case ascertainment and control selection. Give the rationale for the choice of cases and controls  *Cross-sectional study*—Give the eligibility criteria, and the sources and methods of selection of participants | 5 | “While it is possible to follow readmissions for a patient, we limited analyses to the delivery hospitalization”  “Deliveries at or beyond 24 weeks gestation with hospital discharges between 10/2015 and 12/2020 were included. The gestational age of 24 weeks was chosen because it is traditionally considered the limit of viability. Pregnant patients were identified based on procedural coding with the International Classification of Diseases 10th revision, Clinical Modification (ICD-10-CM) (Supplemental Table)” |
|  |  | (*b*) *Cohort study*—For matched studies, give matching criteria and number of exposed and unexposed  *Case-control study*—For matched studies, give matching criteria and the number of controls per case | N/A | N/A |
| Variables | 7 | Clearly define all outcomes, exposures, predictors, potential confounders, and effect modifiers. Give diagnostic criteria, if applicable | 5-6 | “Weeks of gestation was determined using the Z3A.xx series of codes. Diagnosis of HCV was identified for each patient using the ICD-10-CM codes for carrier of viral hepatitis C (Z22.52), chronic viral hepatitis C (B18.2), unspecified viral hepatitis C without hepatic coma (B19.20), or unspecified viral hepatitis C with coma (B19.21). Diagnosis of ICP was determined by whether patients had ICD-10 codes for both liver and biliary tract disorder in pregnancy (O26.6*) and obstruction of bile duct (K83.1). We consulted with an experienced inpatient obstetrics coder at our institution in developing these criteria.”  “The primary outcome for this analysis was occurrence of severe maternal morbidity (SMM) during the delivery hospitalization, based on criteria and the ICD-10 codes published by the Centers for Disease Control and Prevention (CDC)[11]. Our secondary outcomes included acute respiratory distress, acute kidney injury, sepsis, GDM, cesarean delivery, preterm birth defined as birth at less than 37 weeks gestational age, and hospital length of stay” |
| Data sources/ measurement | 8* | For each variable of interest, give sources of data and details of methods of assessment (measurement). Describe comparability of assessment methods if there is more than one group | 4-5 | “This retrospective cohort study was conducted using the Nationwide Readmissions Database (NRD), from the United States Agency for Healthcare Research and Quality’s Healthcare Cost and Utilization Project. The NRD is an all-payor administrative database designed to represent short-stay inpatient admissions in the United States. A total of 28 states participated in the NRD in 2020, including 59.7% of the population and 58.7% of hospitalizations.”  “The data include patient demographics, hospital characteristics, International Classification of Diseases Diagnosis and Procedure codes, discharge disposition, length of stay, and inpatient charges” |
| Bias | 9 | Describe any efforts to address potential sources of bias | 5 | “Pregnant patients were identified based on procedural coding with the International Classification of Diseases 10th revision, Clinical Modification (ICD-10-CM) (Supplemental Table). The starting date was selected given this was when ICD-10 was adopted for clinical use in the United States.”  “We consulted with an experienced inpatient obstetrics coder at our institution in developing these criteria.” |
| Study size | 10 | Explain how the study size was arrived at | 5 | “The NRD is an all-payor administrative database designed to represent short-stay inpatient admissions in the United States. A total of 28 states participated in the NRD in 2020, including 59.7% of the population and 58.7% of hospitalizations. Weighting and stratification variables are provided in the NRD dataset to allow estimating national rates of hospitalizations using the NRD sample.”  “Deliveries at or beyond 24 weeks gestation with hospital discharges between 10/2015 and 12/2020 were included.” |

Continued on next page

| Quantitative variables | 11 | Explain how quantitative variables were handled in the analyses. If applicable, describe which groupings were chosen and why | 6 | “The NRD datasets includes weighting and stratification variables to allow for estimating national rates of hospitalizations. For comparing demographics, comorbidities, hospital characteristics, and SMM in each patient cohort, weighted linear regressions were used for continuous variables and weighted chi-square testing was used for binary and categorical variables as appropriate.” |
| --- | --- | --- | --- | --- |
| Statistical methods | 12 | (*a*) Describe all statistical methods, including those used to control for confounding | 6 | “Weighted logistic (all outcomes except length of stay) and negative binomial (length of stay) regression analyses were used to evaluate the association between HCV and ICP status and outcomes. Models were adjusted for age, primary payer, median household income by ZIP code, hospital type and size, year, discharge quarter, and clinical co-morbidities as identified by the expanded obstetric comorbidity index [12].” |
|  |  | (*b*) Describe any methods used to examine subgroups and interactions | 6 | “We assessed for synergistic or antagonistic effects by including an interaction term between ICP and HCV, which was retained in the final statistical models if it was statistically significant for the given outcome” |
|  |  | (*c*) Explain how missing data were addressed | 6 | “Missing values were only present for the ZIP code income (0.4%) and primary payer (0.06%) variables; given the low prevalence of missing values and complications of a weighted dataset modal value imputation was performed.” |
|  |  | (*d*) *Cohort study*—If applicable, explain how loss to follow-up was addressed  *Case-control study*—If applicable, explain how matching of cases and controls was addressed  *Cross-sectional study*—If applicable, describe analytical methods taking account of sampling strategy | 6 | “The NRD datasets includes weighting and stratification variables to allow for estimating national rates of hospitalizations. For comparing demographics, comorbidities, hospital characteristics, and SMM in each patient cohort, weighted linear regressions were used for continuous variables and weighted chi-square testing was used for binary and categorical variables as appropriate” |
|  |  | (*e*) Describe any sensitivity analyses | N/A | N/A |
| Results | | | | |
| Participants | 13* | (a) Report numbers of individuals at each stage of study—eg numbers potentially eligible, examined for eligibility, confirmed eligible, included in the study, completing follow-up, and analysed | 7, Figure 1 | “A total of 10,040,850 deliveries, which after weighting corresponded to 18,712,085 deliveries nationwide on or after 24 weeks gestation, were identified (Figure 1).”  See CONSORT diagram (Fig 1) |
|  |  | (b) Give reasons for non-participation at each stage | Figure 1 | See CONSORT diagram (Fig 1) |
|  |  | (c) Consider use of a flow diagram | Figure 1 | See CONSORT diagram (Fig 1) |
| Descriptive data | 14* | (a) Give characteristics of study participants (eg demographic, clinical, social) and information on exposures and potential confounders | 7, Table 1 | “Patients differed significantly in multiple factors (Table 1). Among the most prominent, substance use disorder was present in 69.2% of patients with HCV and 57.9% of patients with both HCV and ICP, but only in 4.6% of patients with ICP only and 6.3% of patients with neither disease. Similarly, tobacco use disorder was a comorbidity in 52.4% of patients with HCV and 42.8% of patients with both HCV and ICP, and only 3.8% in patients with ICP only and 5.3% of patients with neither disease. HIV was present in 0.7% of patients with HCV only and 0.8% of patients with both HCV and ICP, but only present in 0.1% of both patients with ICP only and neither disease.” |
|  |  | (b) Indicate number of participants with missing data for each variable of interest | Table 1 | “Missing values in Zip Code Income Quartile (44394 observations), and Primary Payor (6638 observations)” |
|  |  | (c) *Cohort study*—Summarise follow-up time (eg, average and total amount) | N/A | N/A |
| Outcome data | 15* | *Cohort study*—Report numbers of outcome events or summary measures over time |  |  |
|  |  | *Case-control study—*Report numbers in each exposure category, or summary measures of exposure |  |  |
|  |  | *Cross-sectional study—*Report numbers of outcome events or summary measures | 7, Table 1 | “Among the weighted estimate of deliveries, 45,368 (0.5%) had HCV only; 84,582 (0.8%) had ICP only; and 1,967 had both HCV and ICP (<0.1%).”  See Table 1 |
| Main results | 16 | (*a*) Give unadjusted estimates and, if applicable, confounder-adjusted estimates and their precision (eg, 95% confidence interval). Make clear which confounders were adjusted for and why they were included | 7-8, Figure 1, Figure 2 | See fig 1, fig 2  “When adjusted regression models were run…the only statistically significant interaction terms were with GDM (OR 1.25, 95% CI 1.06, 1.48) and length of stay (OR 1.14, 95% CI 1.06-1.23)”  “The regression models were repeated, removing all non-statistically significant interaction terms. (Figure 4). … In adjusted analyses, HCV and ICP were each associated with higher odds of SMM (OR 1.53, 95% CI 1.42-1.66), preterm birth (OR 5.09, 95% CI 4.87-5.33), and longer length of stay (rate ratio 1.46, 95% CI 1.35-1.58). HCV was also associated with higher odds of sepsis (OR 1.98, 95% CI 1.63-2.40), cesarean delivery (OR 1.13, 95% CI 1.10-1.16), and lower odds of GDM (OR 0.80, 95% CI 0.76-0.85). ICP was also associated with higher odds of acute kidney injury (OR 1.77, 95% CI 1.52-2.08) and GDM (OR 1.42, 95% CI 1.38-1.46).” |
|  |  | (*b*) Report category boundaries when continuous variables were categorized | N/A | N/A |
|  |  | (*c*) If relevant, consider translating estimates of relative risk into absolute risk for a meaningful time period | N/A | N/A |

Continued on next page

| Other analyses | 17 | Report other analyses done—eg analyses of subgroups and interactions, and sensitivity analyses | 7-8  Figure 3 | “When adjusted regression models were run including interaction terms between HCV and ICP, there was minimal evidence of antagonism or synergy (a greater or lesser effect from the combination of HCV and ICP than expected from the sum of the individual terms; Figure 3). The only statistically significant interaction terms were with GDM (OR 1.25, 95% CI 1.06, 1.48) and length of stay (OR 1.14, 95% CI 1.06-1.23), in which there appeared to be a small amount of synergy (patients stayed longer and had higher prevalence of GDM than expected from the contributions of HCV and ICP together).”  See figure 3 |
| --- | --- | --- | --- | --- |
| Discussion | | | | |
| Key results | 18 | Summarise key results with reference to study objectives | 8 | “This is the first study demonstrating the effects of having both HCV and ICP on maternal and obstetric outcomes. In our study, HCV correlated with an increased odds of SMM, sepsis, cesarean section, preterm birth, longer length of stay, as well as a decreased odds of GDM. ICP was associated with increased odds of SMM, acute kidney injury, GDM, preterm birth, and longer length of stay. There was no evidence of synergy in outcomes for HCV and ICP except for modest effects on GDM and length of stay.” |
| Limitations | 19 | Discuss limitations of the study, taking into account sources of potential bias or imprecision. Discuss both direction and magnitude of any potential bias | 11-12 | “Given that we are limited in what variables we can use by the database, we cannot know the serotype of HCV to stratify outcomes by serotype, or bile acid levels to characterize the severity of ICP. It was also impossible to distinguish whether the patients received treatment for either disease that could have altered outcomes.”  “There are several limitations to administrative databases including reliability of coding data and lack of adequate control variables [27]”  “While the models were adjusted for confounding factors using the Leonard comorbidity index [12], it is possible that HCV in this model is more of a “risk marker” rather than “risk predictor,” as there are many social comorbidities that are strongly correlated with HCV, but undercoded in administrative data.”  “Finally, although ICP is known to increase risk of stillbirth, we were unable to assess this outcome well in these data” |
| Interpretation | 20 | Give a cautious overall interpretation of results considering objectives, limitations, multiplicity of analyses, results from similar studies, and other relevant evidence | 12 | “This study demonstrates that having both HCV and ICP leads to an increased odds of SMM, preterm birth, and increased length of stay when compared to having either HCV, ICP, or both. These data may be useful in counseling patients regarding their increased risk of adverse outcomes when ICP presents in association with HCV vs. ICP alone.” |
| Generalisability | 21 | Discuss the generalisability (external validity) of the study results | 11 | “Our study is strengthened by the large, all-payor nature of the source dataset. Given that we are limited in what variables we can use by the database, we cannot know the serotype of HCV to stratify outcomes by serotype, or bile acid levels to characterize the severity of ICP. It was also impossible to distinguish whether the patients received treatment for either disease that could have altered outcomes. However, the inability to control for these factors likely makes our results generalizable to patients with all types of HCV and severities of ICP.” |
| Other information | |  | | |
| Funding | 22 | Give the source of funding and the role of the funders for the present study and, if applicable, for the original study on which the present article is based | 13 | “Work contained in this manuscript were made possible by the following grants from the National Institutes of Health (K24-AI093969 [VGF]; NIAID (T32-AI100851 [EME]); and TL1-TR002555 [JJF]). Data acquisition was also supported by funding from the Foundation for Women and Girls with Blood Disorders to JJF.” |

*Give information separately for cases and controls in case-control studies and, if applicable, for exposed and unexposed groups in cohort and cross-sectional studies.

**Note:** An Explanation and Elaboration article discusses each checklist item and gives methodological background and published examples of transparent reporting. The STROBE checklist is best used in conjunction with this article (freely available on the Web sites of PLoS Medicine at http://www.plosmedicine.org/, Annals of Internal Medicine at http://www.annals.org/, and Epidemiology at http://www.epidem.com/). Information on the STROBE Initiative is available at www.strobe-statement.org.
