# Supplementary material for: Maternal and neonatal outcomes in patients with hepatitis C and intrahepatic cholestasis of pregnancy: The sum of the parts
Source: PLoS One. 2023 Oct 18;18(10):e0293030. doi: 10.1371/journal.pone.0293030 (PMC10584137; doi:10.1371/journal.pone.0293030)
Supplement: S1 Table — (DOCX) [file pone.0293030.s003.docx]

**SUPPLEMENTARY TABLE**

**Supplementary Table 1.** Reference list of ICD-10 codes used to identify patient characteristics

| **Identification of Delivery Hospitalization**  ICD-10 coding adapted from existing ICD-9 sources  *(ICD-9 from Kuklina, E. V., et al. (2008). "An enhanced method for identifying obstetric deliveries: implications for estimating maternal morbidity." Matern Child Health J 12(4): 469-477.)* | |
| --- | --- |
| **Diagnosis-Related Grouping** | Discharges on or before 9/30/2018: 767, 768, 774, 775 (vaginal delivery); 765, 766 (cesarean deliveries)  On or after 10/1/2018:  768, 796, 797, 798, 805, 806, 807 (vaginal deliveries)  783, 784, 785, 786, 787, 788 (cesarean deliveries) |
| **Outcome of delivery** | Z37.* |
| **Normal delivery** | O80 |
| **Selected delivery procedures** |  |
| Forceps deliveries | 10D07Z3, 10D07Z4, 10D07Z5 |
| Vacuum extraction | 10D07Z6 |
| Other instrumental delivery | 10D07Z8 |
| Internal and combined version and extraction | 10D07Z7 |
| Other manually assisted deliveries | 10E0XZZ |
| Episiotomy | 0W8NXZZ |
| Cesarean delivery | 10D00Z0, 10D00Z1, 10D00Z2 |
| **Exclusions for Early or Abnormal Pregnancies** |  |
| Hydatidform mole | O01.*, D39.2 |
| Other abnormal products of conception | O02.8, O02.9 |
| Ectopic pregnancy | O00.* |
| Abortion/Early Pregnancy Loss | O02.0, O02.1, O03.*, O04.*, Z33.2, O07.*, O08.*, 10A00ZZ, 10A03ZZ, 10A04ZZ, 10A07Z6, 10A07ZW, 10A07ZX, 10A07ZZ, 10A08ZZ |
